# Pycnogonida (Arthropoda) from the Marine Biological Sample Museum in the Third Institute of Oceanography of MNR, with some new records and a checklist of Fujian, China

**DOI:** 10.3897/BDJ.14.e180969

**Published:** 2026-03-16

**Authors:** Jianjia Wang, Yanyan Yang, Dingyong Huang, Chunguang Wang, Feng Zhang

**Affiliations:** 1 The Key Laboratory of Invertebrate Systematics and Application, College of Life Sciences, Hebei University, Baoding, China The Key Laboratory of Invertebrate Systematics and Application, College of Life Sciences, Hebei University Baoding China; 2 Third Institute of Oceanography, Ministry of Natural Resources, Xiamen, China Third Institute of Oceanography, Ministry of Natural Resources Xiamen China; 3 Key Laboratory of Marine Ecological Conservation and Restoration, Ministry of Natural Resources, Xiamen, China Key Laboratory of Marine Ecological Conservation and Restoration, Ministry of Natural Resources Xiamen China; 4 Observation and Research Station of Island and Coastal Ecosystem in the Western Taiwan Straits, Ministry of Natural Resources, Zhangzhou, China Observation and Research Station of Island and Coastal Ecosystem in the Western Taiwan Straits, Ministry of Natural Resources Zhangzhou China; 5 Fujian Provincial Station for Field Observation and Research of Island and Coastal Zone in Zhangzhou, Zhangzhou, China Fujian Provincial Station for Field Observation and Research of Island and Coastal Zone in Zhangzhou Zhangzhou China

**Keywords:** China, Fujian, museum, new record, checklist

## Abstract

**Background:**

Research on sea spiders in China is relatively scarce and there is a severe shortage of regional basic data. In earlier studies, there was a lack of attention given to this small group and museums housed a large number of specimens that had not been identified.

**New information:**

By re-examining the collections of sea spiders in the Marine Biological Sample Museum in the Third Institute of Oceanography of MNR (MBSM), 12 specimens were found to belong to five families, five genera and five species. Amongst them, four species were new records for Fujian, while *Anoplodactylus tubiferus* (Haswell, 1884) was a new record for China. Through reviewing previous studies, a checklist of sea spiders in Fujian was established and this study increased the number of recorded species from 10 to 14.

## Introduction

The research of pycnogonids from the China seas has been limited. Wang et al. (2020) summarised the previous studies and established a checklist containing 41 species recorded in this area. Due to Dr. Bamber's outstanding work (Bamber 1992, Bamber 1997, Bamber 2004) in Taiwan and Hong Kong, these two regions have the largest number of recorded sea spider species in China. As of now, 23 species have been recorded in Taiwan and 10 in Hong Kong (Wang et al. 2020). In comparison, the research on sea spiders in Fujian is extremely scarce. However, as of now, 10 species have been recorded in this area, second only to Taiwan and on par with Hong Kong. It is currently one of the regions with the richest number of species of sea spiders in China. All of this is attributed to the long-term research on macrobenthos conducted by the Third Institute of Oceanography, Ministry of Natural Resources (TIO) in Fujian, especially in Xiamen.

The Marine Biological Sample Museum in the Third Institute of Oceanography of MNR (MBSM) is one of the largest marine biological museums in China, housing over 200,000 samples of marine organisms. In this work, we re-examined the specimens labelled as pycnogonida which were preserved in MBSM. All these specimens had been collected along the coast of Fujian during the surveys for macrobenthos in 2007. All the twelve specimens are identified as five species, including four new recorded species in Fujian and one new in China. In this report, we present descriptions and plates of these specimens and provide a checklist of the currently recorded species in Fujian.

## Materials and methods

**Figure 1.**
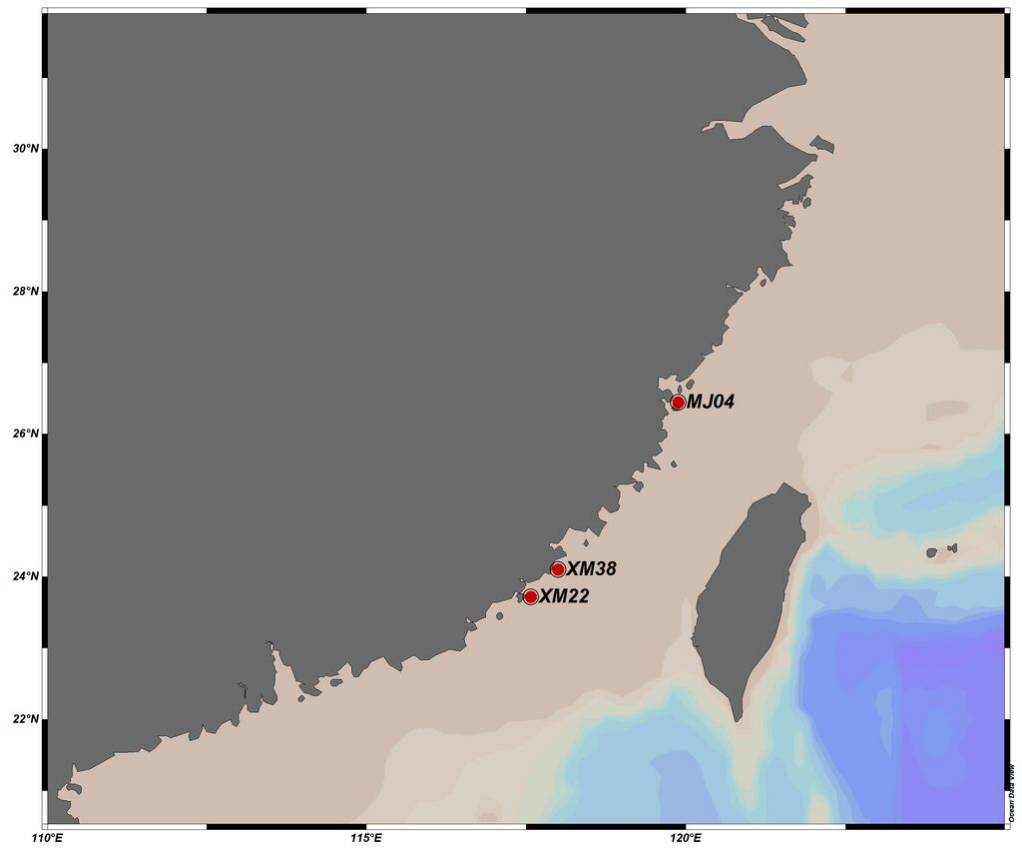
Sampled stations of this study.

All the specimens were collected from the cities of Ningde, Fuzhou and Zhangzhou in Fujian by box corer or trawl during the surveys for macrobenthos in 2007 (Fig. 1, Table 1).

The specimens were preserved in 90% ethanol and stored at the MBSM. The photographs were made with an Auto-montage system on a Leica M205 FA stereomicroscope and z-stacks were created with the LAS software (Version 3.8). Measurements were made axially, dorsally for the trunk, laterally for the proboscis and legs and were given in mm.

## Taxon treatments

### Achelia echinata

Hodge, 1864

CBA783DA-CF91-5815-B654-4F19CF792110

*Achelia echinata Hodge (1864)*: 115, pl. 12, figs. 7–10; Müller (1993): 11–12 [literature]; Kim (2013): 18–21 [literature], figs. 7–8; Munilla and Soler-Membrives (2014): tab. 1 [distribution], 81 [list], 118–121, figs. 43–44, fig. 133D; Lehmann et al. (2014): 165, figs. 2, 5–8; Lee 2020: 30 [key], 40–43 [literature], figs. 12–13 [distribution]; Miyazaki (2022): 2–3 [literature, list]; Lucena and Christoffersen (2025): e109857 [literature], fig. 2.
*Ammothea fibulifera Dohrn (1881)*: 141–145, 15, 49, 51, 81, pl. 4, figs. 1–22.
Ammothea echinata – Sars (1891): 120–124, pl. 13: figs. 1a–m.
Ammothea (Achelia) echinata – Bouvier (1923): 52 [key], 55.
Ammothea echinata orientalis Losina-Losinsky (1933): 55–57, 77–78, figs. 7–8.
Ammothea (Achelia) echinata var. sinensis Lou (1936): 19–30, pls. II–IV, figs. 7–9.
Ammothea (Achelia) echinata var. japonica Ohsima (1936): 865 [literature].
Achelia echinata nasuta Marcus (1940): 84, 128.
Achelia echinata orientalis Hedgpeth (1949): 318.
*Achelia echinata sinensis Nesis (1967)*: 249.

#### Materials

- **Type status:** Other material. **Occurrence:** individualID: TIO2007XM22.001; individualCount: 1; sex: female; lifeStage: adult; reproductiveCondition: with eggs; occurrenceID: 40379966-422E-5AF4-A57D-3519C8B2F1E5; **Taxon:** scientificName: *Achelia echinata* Hodge,1864; **Location:** island: Dongmenyu Islet; country: China; stateProvince: Fujian; verbatimDepth: 36 m; verbatimLatitude: 23.72 N; verbatimLongitude: 117.57 E; **Identification:** identifiedBy: Jianjia Wang; **Event:** samplingProtocol: Box Corer; year: 2007; month: 01; day: 25; habitat: sand

#### Distribution

This species is widely distributed in the Northern Hemisphere, with a depth range of 0 to 300 m (Müller 1993). The previous records in China included Shandong (Qingdao) and Hong Kong (Cape d'Aguilar) (Lou 1936, Bamber 1997). This study extends the distribution of this species in China to Fujian and the depth is consistent with previous records.

#### Remarks

The research history of this species is quite complex, with numerous synonyms and sub-species. This situation might be attributed to Hodge’s (Hodge 1864) initially imperfect description and illustrations, as well as the failure to specify the type specimen, which prevented the re-examination and the provision of more detailed description and illustrations. Our specimens which had a fractured proboscis and ovigers agreed generally with the description and illustrations given by Hodge (1864), Kim (2013), *Lehmann et al. (2014)* and Munilla and Soler-Membrives (2014) by having pipette-shaped proboscis, paired tubercles on the lateral processes and tubercles on the leg articles, while the heel spines are either two or four, which is slightly different from the usual three spines (Fig. 2A and B). Compared to the descriptions and illustrations provided by Lou (1936) and Lee (2020), our specimens have relatively shorter chelifores. Furthermore, in contrast with the description and illustrations provided by Sars (1891) and Lucena and Christoffersen (2025), our specimen presents a more typical pipette-like proboscis, while the distal contraction part of the proboscis of the former is shorter and less distinct and the overall shape is more spindle-shaped.

Miyazaki (2022) briefly summarised the previous research of *A. echinata* and *A. japonica* and indicated that more investigations were still needed to confirm the validity of *A. japonica*, especially considering Ortmann’s (Ortmann 1890) imperfect description. Therefore, in Miyazaki’s (Miyazaki 2022) study, “all records are tentatively collected in *Achelia echinata*”. In Ortman's (Ortmann 1890) description, the thick proboscis and extremely short chelifores of the specimen were significantly different from those of *A. echinata*. This was the important reason why Utinomi (Utinomi 1954, Utinomi 1959) classified it as an independent species. Therefore, further investigation and research on *A. japonica* is necessary, but for now, it should be retained as an independent species. At least, it should not be simply grouped together with *A. echinata*. Moreover, classifying these subspecies (Miyazaki 2022, Bamber et al. 2025), *A. echinata nasuta* Marcus, 1940 (originally *Ammothea echinata orientalis* Losina-Losinsky, 1933) and *A. echinata sinensis* (Ammothea (A.) echinata var. sinensis) (Lou, 1936), which have typical *A. echinata* characteristics in *A. japonica* is also questionable.

### Paranymphon spinosum

Caullery, 1896

B9B89E9C-2620-5634-88F0-E26C4DFF4893

Paranymphon spinosum Caullery (1896): 361-362, pl. 12 figs. 1–6; Müller (1993): 73 [literature]; Kim (2013): 40–43 [literature], figs. 20–21; Munilla and Soler-Membrives (2014): tab. 1 [distribution], 81 [list], 86–89 [key], figs. 63–64, figs. 134C–D; Lee (2020): 93 [key], 96–99 [literature], figs. 41–42 [distribution]; Wang et al. (2020): 363 [literature], fig. 1 [distribution], fig. 2i, tab. 1; Miyazaki (2022): 6–7 [literature, list].

#### Materials

- **Type status:** Other material. **Occurrence:** individualID: TIO2007XM22.004; individualCount: 1; sex: male; lifeStage: adult; reproductiveCondition: with larvae; occurrenceID: 0AE30259-A5E8-50A6-85B4-AA10FE1AEB13; **Taxon:** scientificName: *Paranymphon spinosum* Caullery, 1896; **Location:** island: Dongmenyu Islet; country: China; stateProvince: Fujian; verbatimDepth: 30 m; verbatimLatitude: 23.72 N; verbatimLongitude: 117.57 E; **Identification:** identifiedBy: Jianjia Wang; **Event:** samplingProtocol: Trawl; year: 2007; month: 10; day: 11

#### Distribution

This species is widely distributed in the North Atlantic and West Pacific, with a depth range of 0.6 to 2300 m (Müller 1993, Munilla and Soler-Membrives 2014, Wang et al. 2020). The previous records in China included the East China Sea (28.41°N 123.67°E, 79 m depth) and Xiamen (Fujian) (Wang et al. 2020). This study extends the distribution of this species in China and West Pacific southwards to Zhangzhou (Fujian), with the depth being consistent with previous records.

#### Remarks

This genus currently consists of only four species and they are quite easy to distinguish. Even for *P. spinosum* and *P. magnidigitatum* (Hong and Kim (1987)) that are very similar, they can be separated, based on the size of chela fingers and the number of dorsal tubercles on the first coxa (Wang et al. 2020), (Fig. 3A, B and C).

### Nymphon akanei

Nakamura & Child, 1983

1E9F57B6-83C3-5358-891D-6A94993B6DF5

*Nymphon akane Nakamura and Child (1983)*: 54, fig. 19; Müller (1993): 166 [literature].
Nymphon akanei – Nakamura and Child (1991): 42 [literature]; Bamber (1992): 196–197, fig. 3; Bamber (1997): 153 [text], 155 [key]; Kim (2013): 76–78 [key, literature], figs. 39; Lee (2020): 154–157 [key, literature], figs. 78–79 [distribution]; Miyazaki (2022): 13 [literature, list].

#### Materials

- **Type status:** Other material. **Occurrence:** recordedBy: TIO2007XM22.002; individualCount: 1; sex: male; lifeStage: adult; occurrenceID: 24DAF970-63AC-5894-8257-D70AD2B21381; **Taxon:** scientificName: *Nymphon akanei* Nakamura & Child, 1983; **Location:** island: Dongmenyu Islet; country: China; stateProvince: Fujian; verbatimDepth: 36 m; verbatimLatitude: 23.72 N; verbatimLongitude: 117.57 E; **Identification:** identifiedBy: Jianjia Wang; **Event:** samplingProtocol: Box Corer; year: 2007; month: 1; day: 25; habitat: sand

#### Distribution

This species is distributed in Japan, Korea and China, with a depth range of 7 - 30 m (Bamber 1992, Müller 1993, Lee 2020, Miyazaki 2022). Bamber (1997) has temporarily designated a juvenile specimen collected in Lantau of Hong Kong as belonging to this species. The specimen collected in Dongmenyu Islet of Fujian in this study is the definitive record of this species in China, with the depth range extending up to 36 m.

#### Remarks

The currently recorded species of the genus *Nymphon* in China include *N. akanei*, *N. japonicum* Ortmann 1890 and *N. polyglia* Bamber 2004 (Wang et al. 2020). Li (2003) included *N. japonicum* in his book which systematically discussed the macrobenthos in the continental shelf and adjacent waters of China and indicated that this species is distributed in the Yellow Sea and the East China Sea. Our specimen can be easily distinguished from *N. japonicum*, based on the significantly smaller size, relatively shorter neck, significantly fewer teeth of chelae, shorter tarsus and longer auxiliary claws. Bamber (2004) established *N. polyglia* which is currently only found in Taiwan. Compared with this endemic species, our specimen has a smaller trunk, significantly shorter main articles of legs, longer propodus and auxiliary claws. The more obvious characteristic of *N. polyglia* is the presence of multiple cement gland tubes on the ventral side of both femur and first tibia, while no obvious cement gland openings were found in our specimen.

The species of *N. soyoi* Utinomi 1955, *N. benthos* Hedgpeth 1949, *Nymphon macronyx* Sars 1877 and *N. micronyx* Sars 1888 are closer to this present species (Nakamura and Child 1983, Lee 2020), but the latter has distinct identifying features such as fewer teeth on the chelae fingers, long propodus and long auxiliary claws. Just as Kim (2013) concluded, a combination of characters can easily distinguish *N. akanei* from other species in this genus.

The fourth leg on the left side (detached) is significantly shorter than the others (Fig. 3D). It is speculated that this might be the result of a fracture followed by regeneration due to being preyed upon or other reasons or it could be a case of abnormal development.

### Propallene longiceps

(Böhm, 1879)

F23A13E5-C9E3-517A-8064-2C5C84EA4BDA

Pallene longiceps Böhm (1879): 59–60; Ohshima (1933): 212, figs. 1–6.
Propallene longiceps – Schimkewitsch (1909): 11–13, fig. 3; Stock (1975): 90–91 [literature], 94 [key], figs. 1–20; Müller (1993): 129–130 [literature]; Kim (2013): 73–75 [literature], figs. 37–38; Lee (2020): 145–148 [literature], figs. 71–72 [distribution]; Miyazaki (2022): 13 [literature, list].

#### Materials

- **Type status:** Other material. **Occurrence:** individualID: TIO2007XM38.001; individualCount: 1; sex: female; lifeStage: adult; occurrenceID: 2042BA60-A03D-5473-84C9-FD641443C34F; **Taxon:** scientificName: *Propallene longiceps* (Böhm, 1879); **Location:** country: China; stateProvince: Fujian; locality: Qianhu Bay; verbatimDepth: 21 m; verbatimLatitude: 24.10 N; verbatimLongitude: 118.00 E; **Identification:** identifiedBy: Jianjia Wang; **Event:** samplingProtocol: Box Corer; year: 2007; month: 4; day: 5; habitat: mud
- **Type status:** Other material. **Occurrence:** individualID: TIO2007MJ04.002; individualCount: 5; sex: male; lifeStage: adult; occurrenceID: 1086260F-F33C-5C11-9291-09046B3AA1AB; **Taxon:** scientificName: *Propallene longiceps* (Böhm, 1879); **Location:** country: China; stateProvince: Fujian; locality: Sansha Bay; verbatimDepth: 33 m; verbatimLatitude: 26.45 N; verbatimLongitude: 119.88 E; **Identification:** identifiedBy: Jianjia Wang; **Event:** samplingProtocol: Box Corer; year: 2007; month: 4; day: 17; habitat: mud

#### Distribution

This species is distributed in Japan and Korea, with a depth range of 6 - 103 m (Müller 1993, Lee 2020, Miyazaki 2022). Huang and Lin (2012) included this species which was recorded in the East Sea of China, while Wang et al. (2020) did not include this species in the checklist as no detailed information was available to verify this record. The specimens (Fig. 4) collected in this study represent a detailed record of this species in China, extending its distribution range from South Korea and Japan to the southern West Pacific, with a distribution depth consistent with the previous records.

### Anoplodactylus tubiferus

(Haswell, 1884)

9AF2F9C5-7779-59D4-AAD0-51B056D50D61

*Phoxichilidium tubiferum Haswell (1884)*:1032, pl. 57, figs. 1–5.
*Anoplodactylus tubiferus – Cole (1904)*: 288; Müller (1993): 247 [literature]; Stock (1994): 67 [literature]; Arango (2003): 2747 [key], 2755 [literature], 2757; Lee (2020): 186 [key], 206–211 [literature], figs. 105–106 [distribution]; Miyazaki (2022): 20–21 [literature].
Anoplodactylus pulcher Carpenter (1907): 97–98, pl. 12, figs. 13–19.
Anoplodactylus stylops Loman (1908): 71, pl. 11, figs. 20–24.

#### Materials

- **Type status:** Other material. **Occurrence:** individualID: TIO2007MJ04.001; individualCount: 3; sex: 1 male, 2 female; lifeStage: adult; occurrenceID: 9C58C538-7068-574D-913D-2F745FF169FC; **Taxon:** scientificName: *Anoplodactylus tubiferus* (Haswell, 1884); **Location:** verbatimLocality: Sansha Bay; verbatimDepth: 34 m; verbatimLatitude: 26.45 N; verbatimLongitude: 119.88 E; **Identification:** identifiedBy: Jianjia Wang; **Event:** samplingProtocol: Box Corer; year: 2007; month: 1; day: 7; habitat: mud
- **Type status:** Other material. **Occurrence:** individualID: TIO2007XM22.003; individualCount: 1; sex: 1 male; lifeStage: adult; occurrenceID: 2B4B6ACD-742A-5EAB-A3CE-4602F1EC2612; **Taxon:** scientificName: *Anoplodactylus tubiferus* (Haswell, 1884); **Location:** island: Dongmenyu Islet; country: China; stateProvince: Fujian; verbatimDepth: 30 m; verbatimLatitude: 23.72 N; verbatimLongitude: 117.57 E; **Identification:** identifiedBy: Jianjia Wang; **Event:** samplingProtocol: Trawl; year: 2007; month: 10; day: 11

#### Description

TIO2007MJ04.001 (male): Trunk extremely slender, not segmented; cephalic segment short, slightly contracted in the middle, proximal part sub-triangular. Lateral processes elongated and columnar, separated by about three times their diameters, with long dorsodistal setae arranged in a semi-circular shape. Ocular tubercle located at anterior margin of cephalic segment; slender cylindrical shape, with a length approximately 6 times its base diameter; four eyes located at the top, with one pair on each side. Proboscis approximately 2/5 length of the trunk, tube-shaped, slightly contracted in the middle. Abdomen long and slender, tapering distally, horizontally backwards, without basal articulation. Chelifore 2-segmented; scape slender and rod-shaped, about 4/5 length of the proboscis, dorsally with long setae; chela about one-third of the scape; fingers slender, distally curved, peripherally with setae. Ovigers slender, six-articled; the third article longest, appears to be segmented close to the proximal end; the fourth article slightly curved and slightly longer than the fifth article; the sixth article extremely short, about 1/4 the length of the fifth article. The length of the second pair of legs 4.72 mm; major articles with long dorsal setae; first coxae with long dorsodistally setae; second coxae with long dorsal setae; cement glands slender tube-shaped, long, irregularly curved, located at proximal 2/5 of the femur; propodus bent proximally, with two heel spines; ten sole spines, distally flat and bent towards the main claw; main claw strong, slightly curved distally, about 3/4 the length of the propodus; auxiliary claws extremely weak and not obvious, about 1/20 the length of the main claw (Fig. 5E and F).

#### Distribution

This species is widely distributed in the Indo-West Pacific Region, including Japan, South Korea, Philippines, Indonesia, Australia, India, Maldives, Madagascar, Seychelles, Persian Gulf and Aden Gulf, with a depth range of 2 - 235 m (Müller 1989, Müller 1993, Arango 2003, Lee 2020, Miyazaki 2022). The specimens collected in Sansha Bay and Dongmenyu Islet of Fujian in this study are new records of this species in China and the depth is consistent with previous records.

#### Remarks

The species in the genus *Anoplodactylus* are difficult to distinguish, while this species is very distinct with slender body, long setae on lateral processes and unique long cement gland tube (Fig. 5A-E). This species can be easily distinguished from *Anoplodactylus erectus* Cole 1904, *Anoplodactylus glandulifer* Stock 1954, *Anoplodactylus eroticus* Stock 1968 and the two indeterminate species in Taiwan, having the slender habit and the distinct intervals between lateral processes (Fig. 5)

Our specimen is basically consistent with previous descriptions and figures (Haswell 1884, Arango 2003, Lee 2020). Müller (1989) showed the dorsodistally slender tubercles on the femur and first tibia, while the specimens collected in Staples (1982), Lee (2020) and this study have the dorsodistally small tubercles with long setae (Fig. 5E).

## Checklists

### Checklist of sea spiders in Fujian

#### Ammotheidae

EA7C24B6-D5B9-5B6A-99F8-5CA50AA77812

#### Achelia echinata

Hodge,1864

E0693F37-FD92-5D66-A10D-DC57870A25E2

##### Materials

- **Type status:** Other material. **Occurrence:** sex: 1 female; occurrenceID: 40379966-422E-5AF4-A57D-3519C8B2F1E5; **Location:** country: China; stateProvince: Fujian; county: Zhangzhou; locality: Dongmenyu Islet; verbatimElevation: 36 m; **Event:** eventDate: 25/1/2007

##### Distribution

Widely distributed in the Northern Hemisphere.

##### Notes

First record in Fujian, China.

#### Achelia superba

(Loman, 1911)

E5AA7018-8DEE-5D8B-BB25-2E48EF14B2A6

##### Distribution

West Pacific.

##### Notes

Xiamen (Huang 2006).

#### Ammothea hilgendorfi

(Böhm, 1879)

B3AB5755-9EEB-5EED-83FE-DD6967D675F4

##### Distribution

Global distribution.

##### Notes

Pingtan, Fuqing, Xiamen (Wang et al. 1996; Wang et al. 2020).

#### Tanystylum sinoabductus

Bamber, 1992

10ADF39F-1CE9-5D6B-87DD-83D7E85F9B73

##### Distribution

China.

##### Notes

Xiamen (Huang 2006).

#### Ascorhynchidae

12D48E48-46E9-5631-8305-D328F62CE4F1

#### Ascorhynchus auchenicus

(Slater, 1879)

C705720D-457E-57BF-8172-FF019EB55DD6

##### Distribution

Japan, China.

##### Notes

Xiamen (Huang 2006).

#### Paranymphon spinosum

Caullery 1896

59419023-C683-5EAB-AB6C-6F87F24AB087

##### Materials

- **Type status:** Other material. **Occurrence:** sex: 1 male; occurrenceID: 0AE30259-A5E8-50A6-85B4-AA10FE1AEB13; **Location:** country: China; stateProvince: Fujian; county: Zhangzhou; locality: Dongmenyu Islet; verbatimElevation: 30 m; **Event:** eventDate: 11/10/2007

##### Distribution

North Atlantic and West Pacific.

##### Notes

Xiamen (Wang et al. 2020); first record in Zhangzhou, Fujian, China.

#### Nymphonidae

6C77945F-7330-5F5B-8E22-C8D32AB98649

#### Nymphon akanei

Nakamura & Child, 1983

2BD2B104-F96B-52D2-8B74-579E553B222B

##### Materials

- **Type status:** Other material. **Occurrence:** sex: 1 male; occurrenceID: 24DAF970-63AC-5894-8257-D70AD2B21381; **Location:** country: China; stateProvince: Fujian; county: Zhangzhou; locality: Dongmenyu Islet; verbatimElevation: 36 m; **Event:** eventDate: 25/1/2007

##### Distribution

Japan, Korea, China.

##### Notes

First record in Fujian, China.

#### Nymphon

130FB5CC-9A15-5E7D-90AF-01E4DF937713

##### Notes

Xiamen (Huang 2006).

#### Callipallenidae

624C41E2-44CD-5874-9004-BC0C11A87102

#### Callipallene dubiosa

Hedgpeth, 1949

4EBFE14C-5DFC-5D4F-A8B1-0F038A5FE4BA

##### Distribution

Indo-West Pacific.

##### Notes

Ximen (Stock 1954), Hong Kong (Bamber 1997).

#### Propallene longiceps

(Bohm, 1879)

73B3A415-2486-5934-9671-3100327F5E65

##### Distribution

Japan, Korea, China.

##### Notes

First record in Fujian, China.

#### Propallene

0647E92D-12FF-5D68-996C-952B375BDB0D

##### Materials

- **Type status:** Other material. **Occurrence:** sex: 1 female; occurrenceID: 2042BA60-A03D-5473-84C9-FD641443C34F; **Location:** country: China; stateProvince: Fujian; county: Zhangzhou; locality: Qianhu Bay; verbatimElevation: 21 m; **Event:** eventDate: 5/4/2007
- **Type status:** Other material. **Occurrence:** sex: 5 male; occurrenceID: 1086260F-F33C-5C11-9291-09046B3AA1AB; **Location:** country: China; stateProvince: Fujian; county: Ningde; locality: Sansha Bay; verbatimElevation: 33 m; **Event:** eventDate: 17/4/2007

##### Notes

Xiamen (Wang et al. 2020).

#### Phoxichilidiidae

304E87D0-6A92-50B3-9B3F-CFDE297DE441

#### Anoplodactylus eroticus

Stock, 1968

E453675B-BC3F-54F6-884A-0AF70AD91FA0

##### Distribution

India, Hawaii, Brazil, China.

##### Notes

Putian (Wang et al. 2020).

#### Anoplodactylus glandulifer

Stock, 1954

C20F0FBF-F6CF-5D56-AC33-333AD42D18C8

##### Distribution

Widely distributed in the Indo-Pacific and North Atlantic.

##### Notes

Xiamen (Huang 2006).

#### Anoplodactylus tubiferus

(Haswell, 1884)

339613FD-3B4A-5414-A634-39609C8317DF

##### Materials

- **Type status:** Other material. **Occurrence:** sex: 1 male, 2 female; occurrenceID: 9C58C538-7068-574D-913D-2F745FF169FC; **Location:** country: China; stateProvince: Fujian; county: Ningde; locality: Sansha Bay; verbatimElevation: 34 m; **Event:** eventDate: 7/1/2007
- **Type status:** Other material. **Occurrence:** sex: 1 male; occurrenceID: 2B4B6ACD-742A-5EAB-A3CE-4602F1EC2612; **Location:** country: China; stateProvince: Fujian; county: Zhangzhou; locality: Dongmenyu Islet; verbatimElevation: 30 m; **Event:** eventDate: 11/10/2007

##### Distribution

Widely distributed in the Indo-Pacific and West Pacific.

##### Notes

First record in Fujian, China.

## Discussion

### Checklist of sea spiders in Fujian

Similar to other regions in China, research on sea spiders in Fujian has remained relatively limited. However, extensive investigations into macrobenthic fauna have led to the documentation of a considerable number of sea spider species in the area. For instance, Wang et al. (1996) reported *Ammothea hilgendorfi* (Böhm 1879) from fouling organisms in Xiamen Port. Huang (2006) listed six species of sea spiders belonging to six genera and four families from Xiamen Bay, while Li (2010) recorded three species along the coastal zone of Fujian and the western Taiwan Strait. Wang et al. (2020) further expanded the known diversity by discovering and describing four species in Fujian, two of which were newly recorded in China, bringing the total number of sea spider species recorded in the region to ten — second only to Taiwan and comparable to Hong Kong (Table 2).

Recent re-examination of specimens deposited in the MBSM has increased the number of recorded sea spider species in Fujian to fourteen. Based on current species distribution data, the Xiamen-Zhangzhou area appears to be a potential biodiversity hotspot for sea spiders in Fujian.

## Supplementary Material

XML Treatment for Achelia echinata

XML Treatment for Paranymphon spinosum

XML Treatment for Nymphon akanei

XML Treatment for Propallene longiceps

XML Treatment for Anoplodactylus tubiferus

XML Treatment for Ammotheidae

XML Treatment for Achelia echinata

XML Treatment for Achelia superba

XML Treatment for Ammothea hilgendorfi

XML Treatment for Tanystylum sinoabductus

XML Treatment for Ascorhynchidae

XML Treatment for Ascorhynchus auchenicus

XML Treatment for Paranymphon spinosum

XML Treatment for Nymphonidae

XML Treatment for Nymphon akanei

XML Treatment for Nymphon

XML Treatment for Callipallenidae

XML Treatment for Callipallene dubiosa

XML Treatment for Propallene longiceps

XML Treatment for Propallene

XML Treatment for Phoxichilidiidae

XML Treatment for Anoplodactylus eroticus

XML Treatment for Anoplodactylus glandulifer

XML Treatment for Anoplodactylus tubiferus

## Figures and Tables

**Figure 2a.**
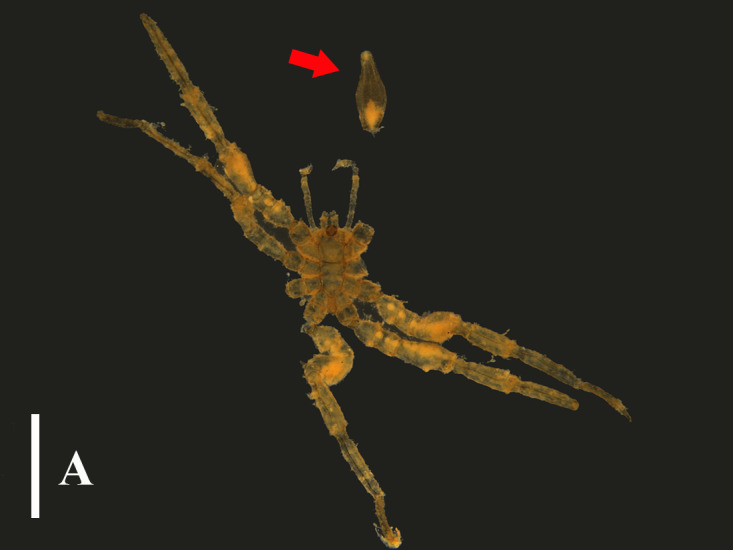
Dorsal view, arrows indicate the broken proboscis. Scale bar = 1 mm;

**Figure 2b.**
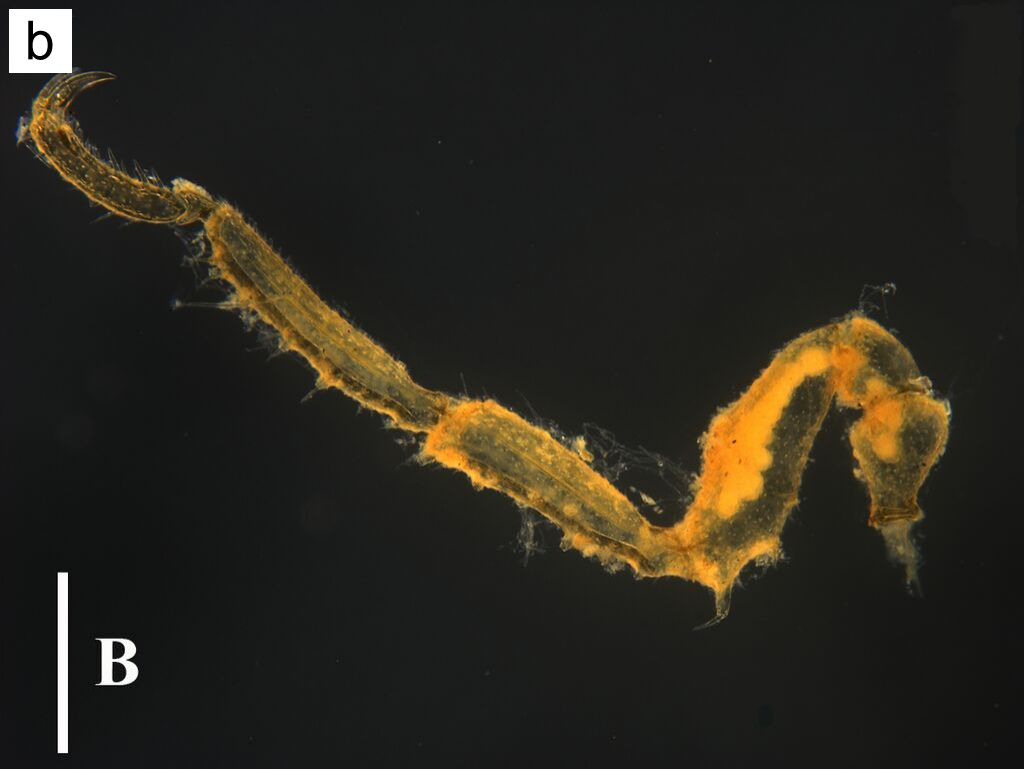
Leg 1. Scale bar = 0.5 mm.

**Figure 3a.**
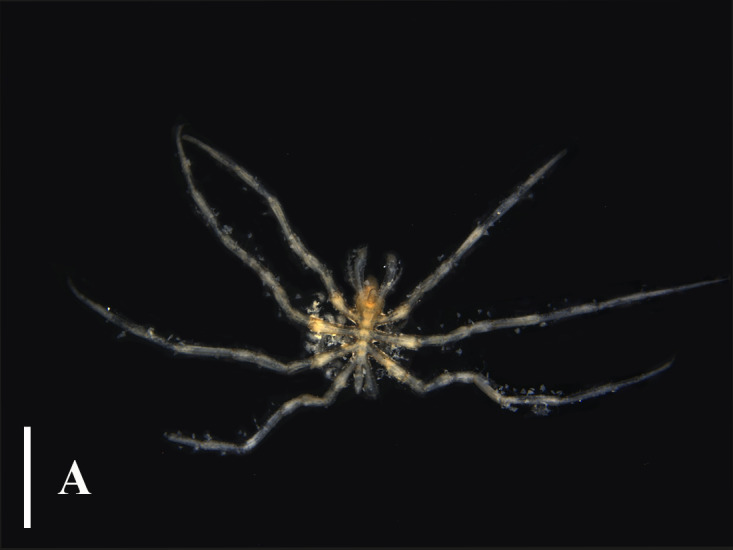
*Paranymphon spinosum* (TIO2007XM22.004), dorsal view. Scale bar = 1 mm;

**Figure 3b.**
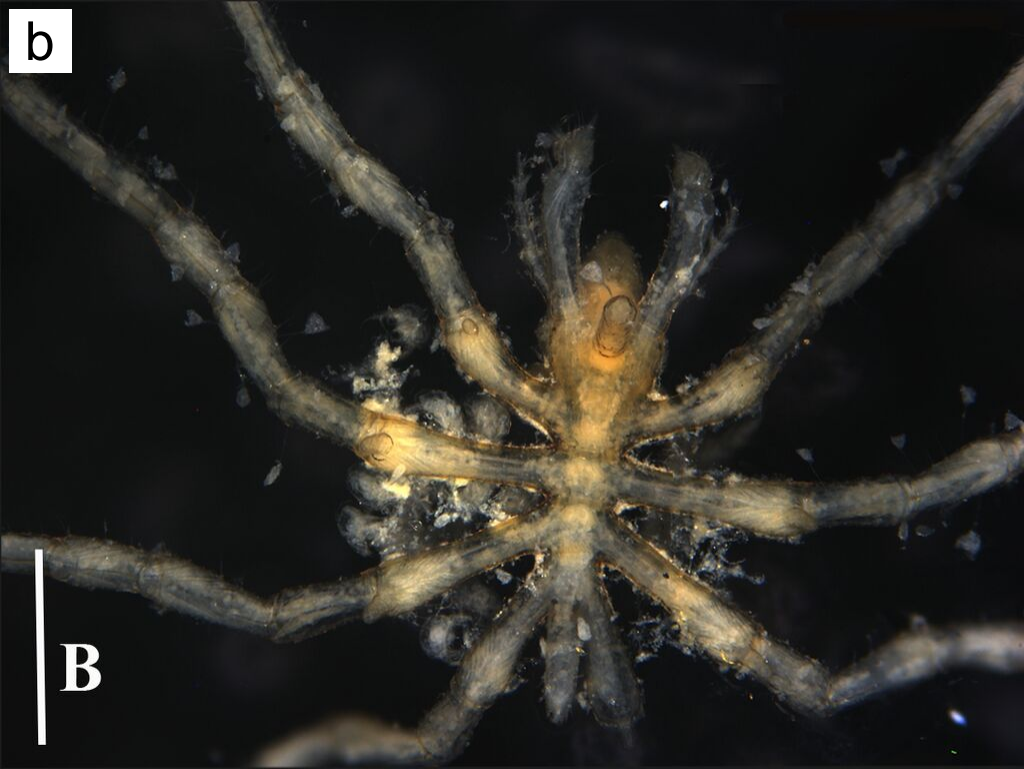
*Paranymphon spinosum* (TIO2007XM22.004), trunk, dorsal view. Scale bar = 0.5 mm;

**Figure 3c.**
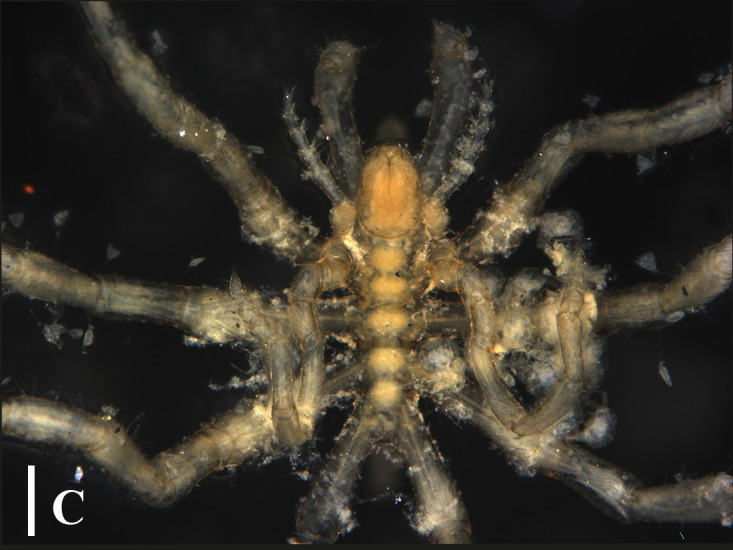
*Paranymphon spinosum* (TIO2007XM22.002), trunk, ventral view. Scale bars = 2 mm;

**Figure 3d.**
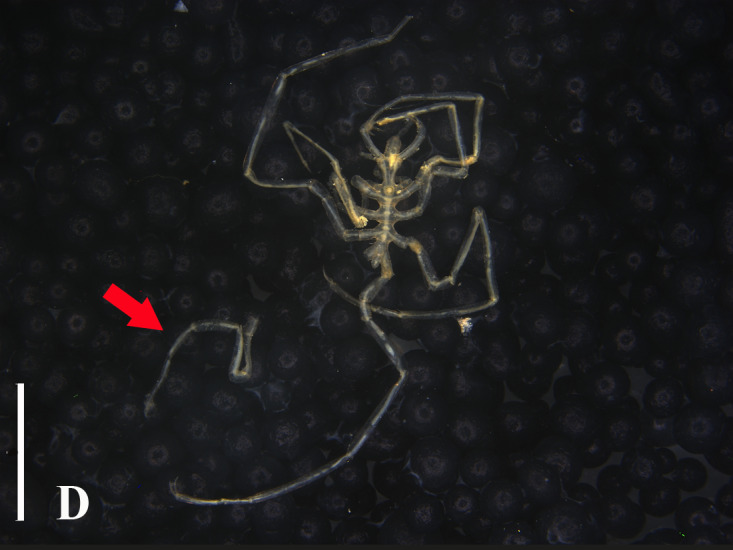
*Nymphon akanei* (TIO2007XM22.002), dorsal view, arrow indicates the abnormal, detached, leg 4. Scale bar = 2 mm;

**Figure 3e.**
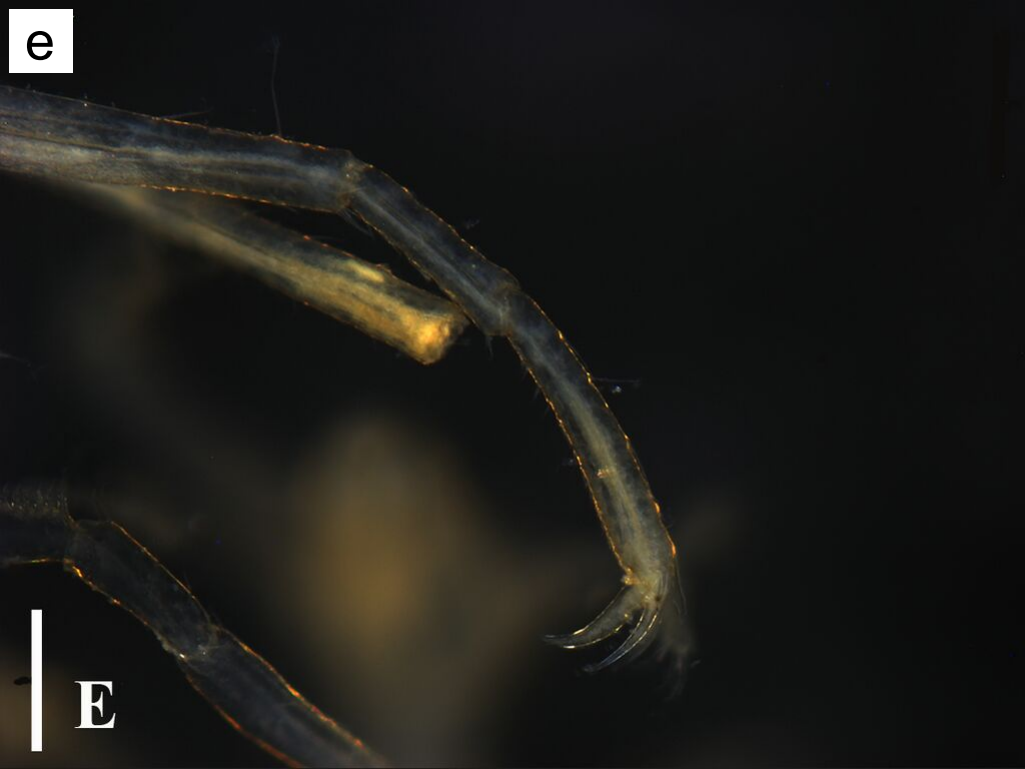
*Nymphon akanei* (TIO2007XM22.002), tarsus, propodus and claw of leg 1, enlarged. Scale bar = 0.2 mm;

**Figure 3f.**
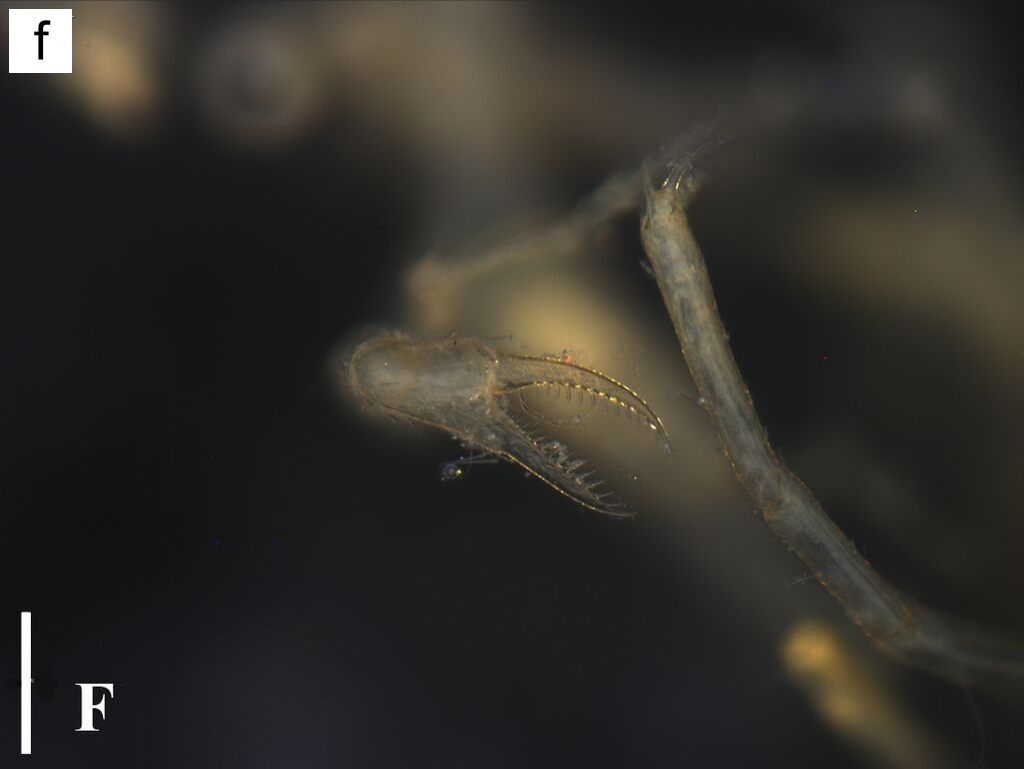
*Nymphon akanei* (TIO2007XM22.002), chela. Scale bar = 0.2 mm.

**Figure 4a.**
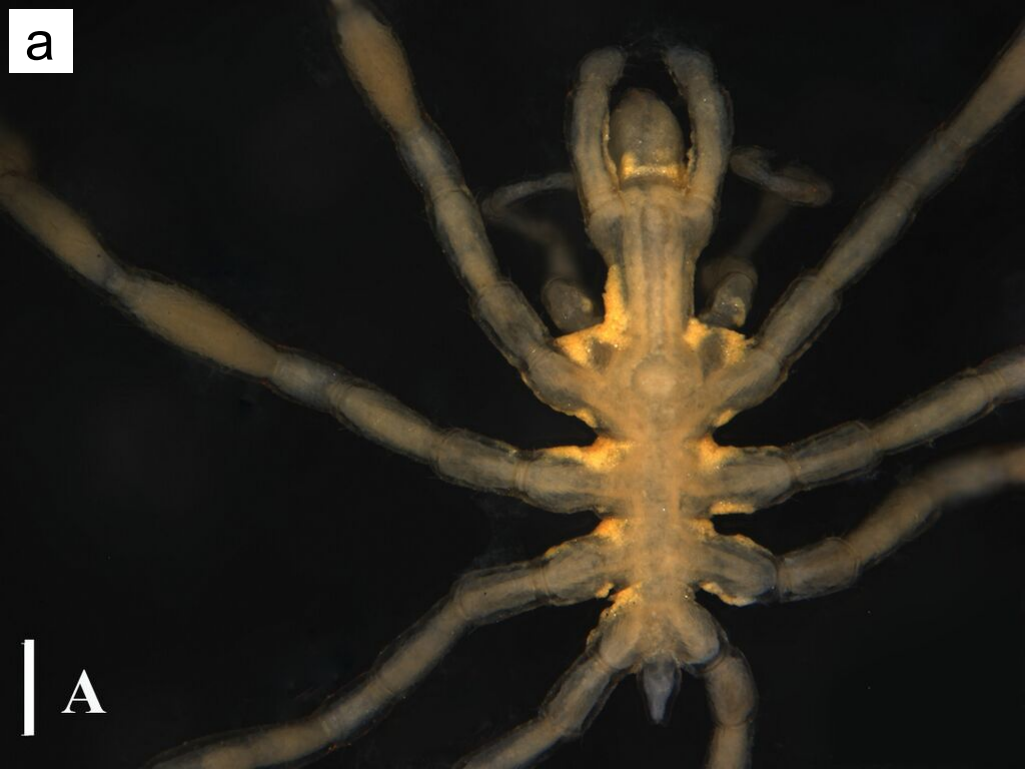
Dorsal view;

**Figure 4b.**
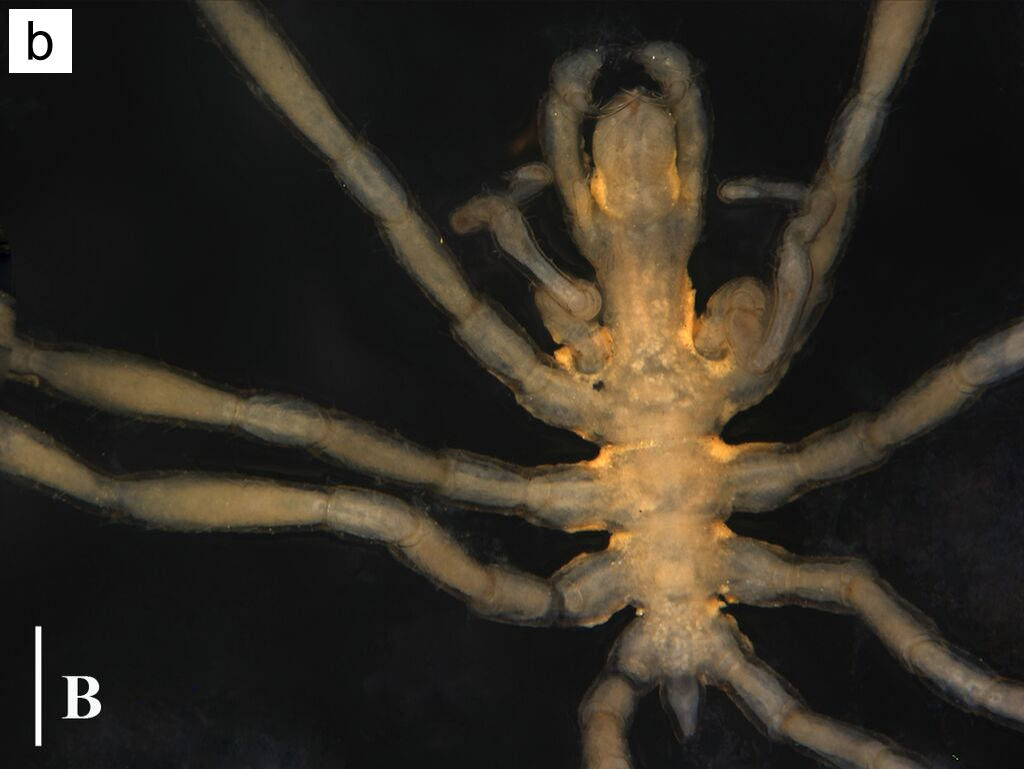
Ventral view.

**Figure 5a.**
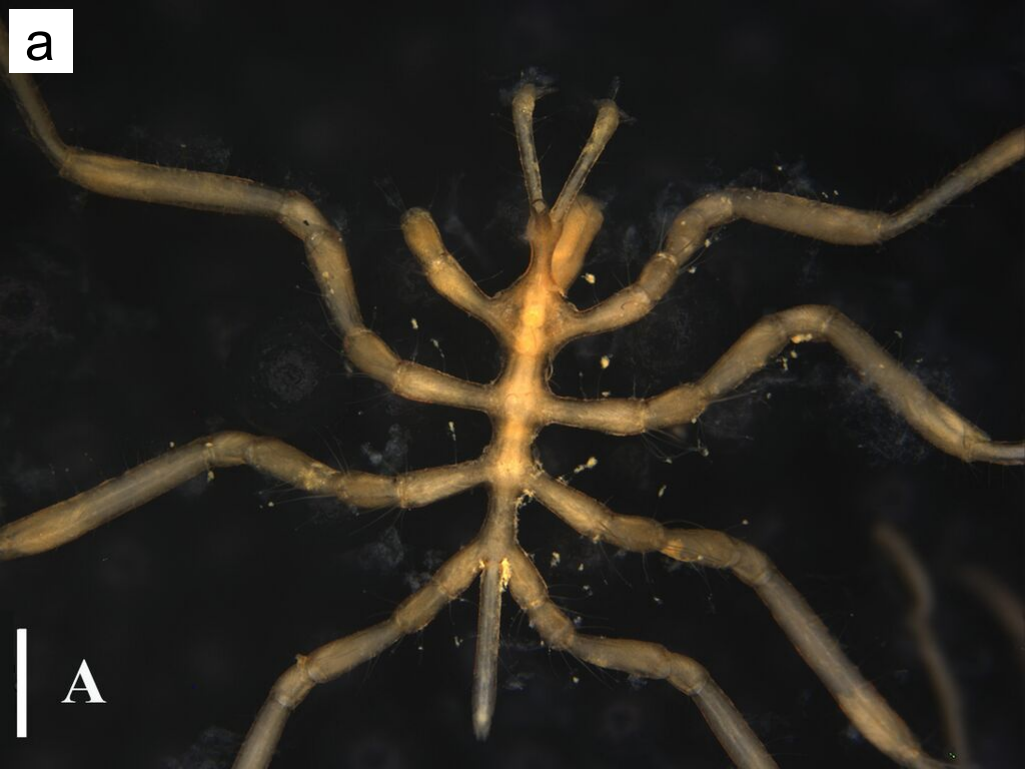
Female (TIO2007MJ04.001), trunk, dorsal view. Scale bar 0.5 mm;

**Figure 5b.**
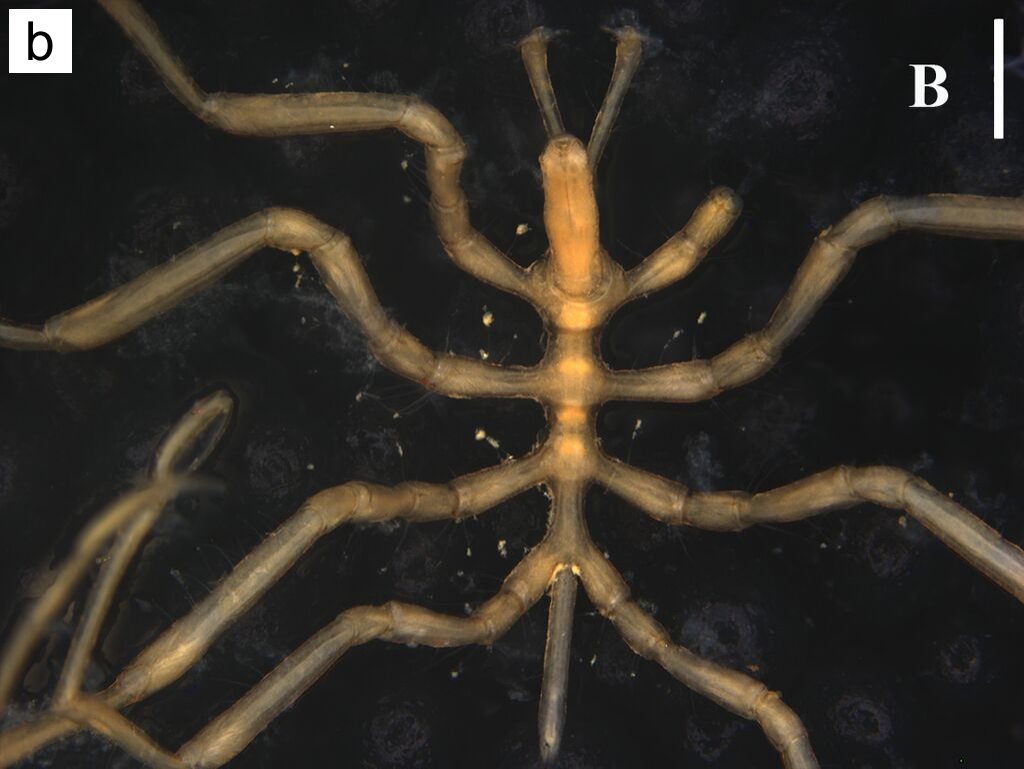
Female (TIO2007MJ04.001), trunk, ventral view. Scale bar 0.5 mm;

**Figure 5c.**
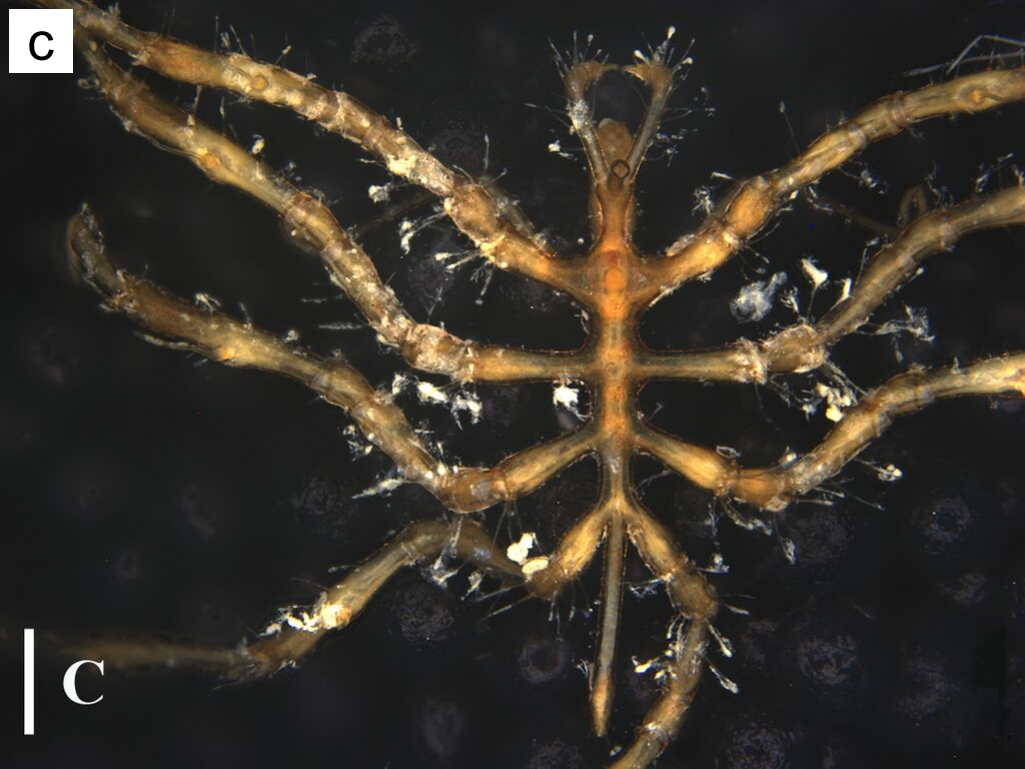
Male (TIO2007MJ04.001), trunk, dorsal view. Scale bar = 0.5 mm;

**Figure 5d.**
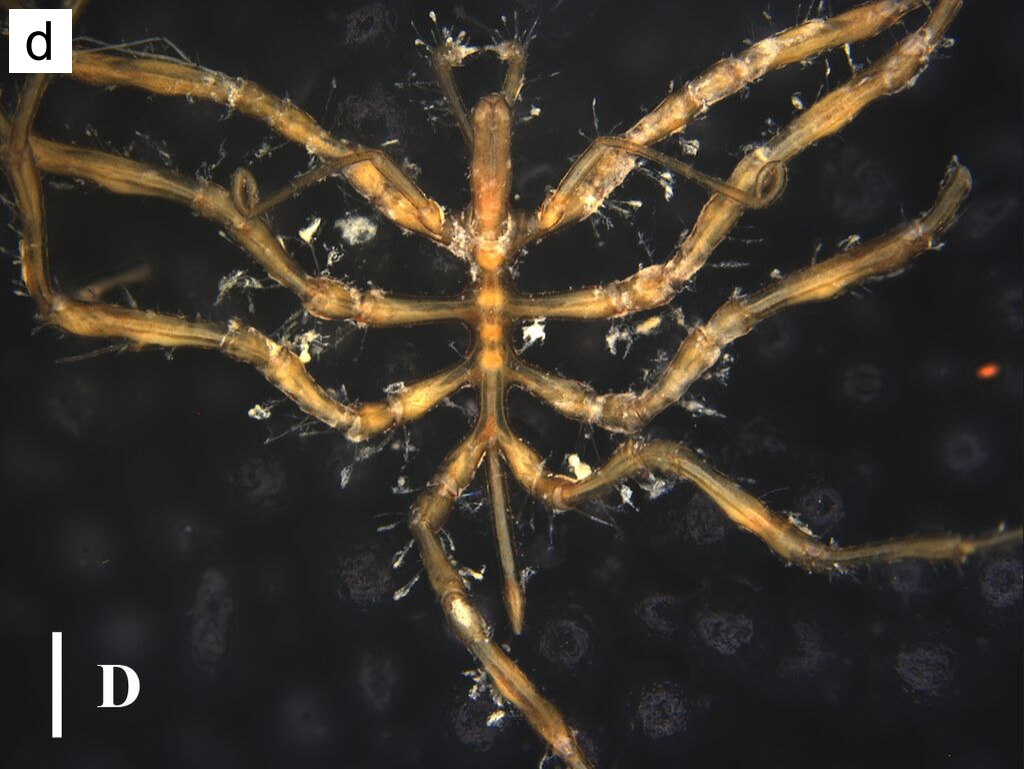
Male (TIO2007MJ04.001), trunk, ventral view. Scale bar = 0.5 mm;

**Figure 5e.**
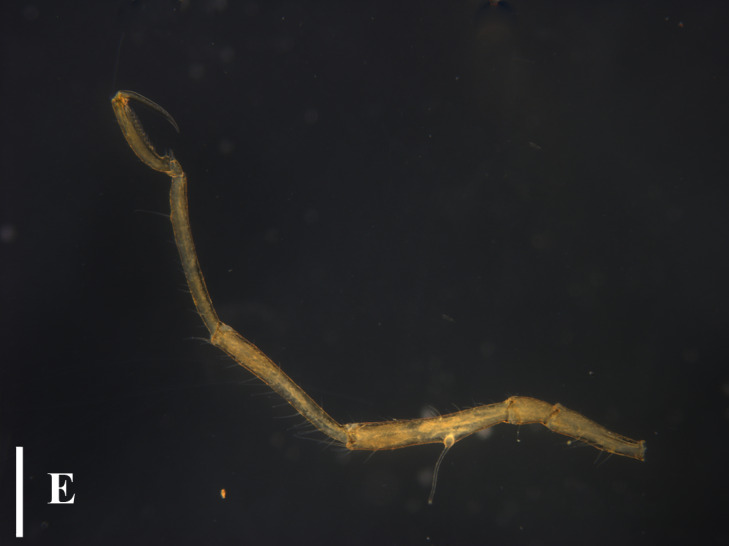
Male (TIO2007MJ04.001), leg 4. Scale bar = 0.5 mm;

**Figure 5f.**
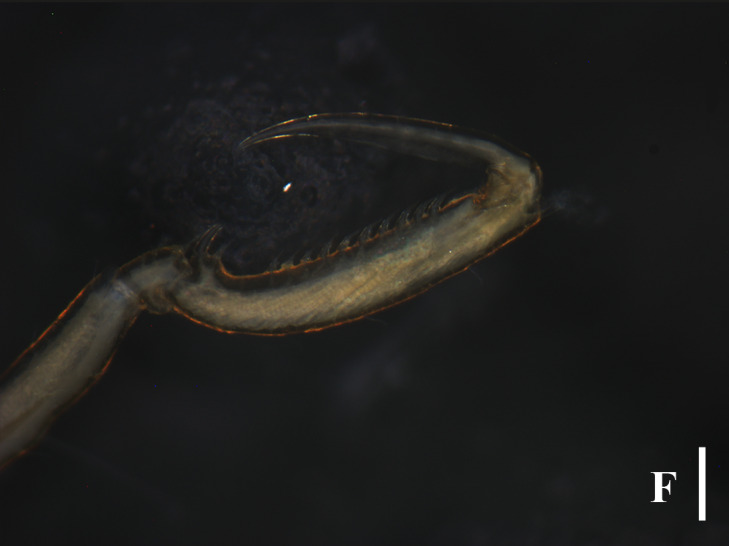
Male (TIO2007MJ04.001), tarsus, propodus and claw of leg 4, enlarged. Scale bar = 0.1 mm.

**Table 1.** Information on sample stations. BC: Box Corer; T: Trawl

Station Number	Location (City)	Longitude (°E)	Latitude (°N)	Depth (m)	Time	Sampling Equipment
MJ04	Ningde	119.88	26.45	34	2007.01.07	BC
XM22	Zhangzhou	117.57	23.72	36	2007.01.25	BC
XM38	Zhangzhou	118.00	24.10	21	2007.04.05	BC
MJ04	Ningde	119.88	26.45	33	2007.04.17	T
XM22	Zhangzhou	117.57	23.72	30	2007.10.11	T

**Table 2.** Checklist of sea spiders in Fujian (the content of this study is in bold).

NO.	Species	Distribution in Fujian	Distribution in other regions of China
1.	***Achelia echinata*** Hodge 1864	**Zhangzhou (Dongmenyu Islet)**	Shandong (Qingdao) , Hong Kong (Cape d'Aguilar)
2.	*Achelia superba* (Loman 1911)	Xiamen	Liaoning, Shandong
3.	*Ammothea hilgendorfi* (Böhm 1879)	Fuzhou (Pingtan, Fuqing), Xiamen	Shallow sea
4.	***Paranymphon spinosum*** Caullery 1896	Xiamen, **Zhangzhou (Dongmenyu Islet)**	Yellow Sea, East China Sea
5.	*Tanystylum sinoabductus* Bamber 1992	Xiamen	Hong Kong
6.	*Ascorhynchus auchenicus* (Slater 1879)	Xiamen	East China Sea
7.	***Nymphon akanei*** Nakamura and Child 1983	**Zhangzhou (Dongmenyu Islet)**	Hong Kong
8.	*Nymphon* sp.	Xiamen	-
9.	*Callipallene dubiosa* Hedgpeth 1949	Xiamen	Hong Kong
10.	***Propallene longiceps*** (Böhm 1879)	**Zhangzhou (Qianhu Bay), Ningde (Sansha Bay)**	East China Sea
11.	*Propallene* species indeterminate	Xiamen	-
12.	*Anoplodactylus eroticus* Stock 1968	Putian	-
13.	*Anoplodactylus glandulifer* Stock 1954	Xiamen	Hong Kong
14.	***Anoplodactylus tubiferus*** (Haswell 1884)	**Zhangzhou (Dongmenyu Islet), Ningde (Sansha Bay)**	-
